# Chondrogenesis, joint formation, and cartilage metabolism

**DOI:** 10.1186/ar3712

**Published:** 2012-03-08

**Authors:** Mary B Goldring

**Affiliations:** 1 The Hospital for Special Surgery, Laboratory for Cartilage Biology, Tissue Engineering Repair & Regeneration Program, Weill Cornell Medical College, New York, NY, USA

## 

Chondrogenesis occurs as a result of mesenchymal cell condensation and chondroprogenitor cell differentiation. The chondrocytes then form the cartilage at the end of the opposing bones with the intervening interzones formed during cavitation or they undergo proliferation, terminal differentiation to chondrocyte hypertrophy, and apoptosis in a process termed endochondral ossification, whereby the hypertrophic cartilage is replaced by bone. A similar sequence of events occurs in the postnatal growth plate and leads to rapid growth of the skeleton. Human adult articular cartilage is a complex tissue of matrix proteins that varies from superficial to deep layers and from loaded to unloaded zones. A major challenge to efforts to repair cartilage by stem cell-based and other tissue engineering strategies is the inability of the resident chondrocytes to lay down new matrix with the same properties as it had when it was formed during development. This is particularly true of the collagen network, which is susceptible to cleavage once the proteoglycans are depleted. Thus, understanding and comparing the mechanisms of cartilage remodeling during development, osteoarthritis (OA), and aging may lead to more effective strategies for preventing cartilage damage and promoting repair. To identify and characterize mediators of cartilage remodeling common to these processes, we are using culture models of primary human and mouse chondrocytes and cell lines and mouse models to manipulate and compare gene expression with complementary approaches. MMP-13, the major type II collagen-degrading collagenase, is regulated by both stress and inflammatory signals that not only contribute to irreversible joint damage (progression) in OA, but importantly, also to the initiation/onset phase, wherein chondrocytes in articular cartilage leave their natural growth - and differentiation-arrested state. We and other investigators have found that there are common mediators of these processes in human OA cartilage and from early through late stages of OA in mouse models, including the surgical models (good matrix with abnormal loading) and the genetic models during aging (bad matrix with normal loading). We are validating our in vitro analyses of the signaling and transcriptional mechanisms that determine the expression and activities of these mediators by in vivo analyses of the consequences of knockout or transgenic overexpression of these genes in mouse models. In current studies, we are examining the epigenetic mechanisms and using proteomics and genomics approaches to map the signaling networks and microRNA targets that impact on gene expression programs during the onset and progression of OA in both human and murine cartilage. Since the chondrocytes in adult human cartilage are normally quiescent and maintain the matrix in a low turnover stated, understanding how they undergo phenotypic modulation and promote matrix destruction and abnormal repair in OA may to lead to identification of critical targets for therapy to block cartilage damage and promote effective cartilage repair.

